# Challenges of Writing Theses and Dissertations in an EFL Context: Genre and Move Analysis of Abstracts Written by Turkish M.A. and Ph.D. Students

**DOI:** 10.3389/fpsyg.2022.925420

**Published:** 2022-06-27

**Authors:** Serdar Sükan, Behbood Mohammadzadeh

**Affiliations:** ^1^Department of Modern Languages, Cyprus International University, Nicosia, Turkey; ^2^ELT Department, Faculty of Education, Cyprus International University, Nicosia, Turkey

**Keywords:** genre, move, abstracts, ELT M.A. thesis, ELT Ph.D. thesis, Hyland model

## Abstract

Writing a thesis or dissertation is a challenging procedure as it is one of the requirements of getting a graduate and postgraduate diploma. Writing an abstract like other parts of a thesis or dissertation has its criterion. For this reason, due to globalism, those abstracts written by non-native English speakers may lack some of the features of the abstract genre and move that must be included. This study examines the moves of M.A. and Ph.D. abstracts written by Turkish students between the 2009 and 2019 academic years on foreign language education at Cyprus International University. The data consisted of 50 abstracts chosen randomly from the ELT department. For the analysis, Hyland’s five-move model has been used. The study results reveal that 40 abstracts did not follow the five moves that Hyland has put forward. Moreover, it can be stated that the absence of some moves in the abstracts may cause restraint for readers to comprehend these studies in terms of communicative purposes.

## Introduction

In recent years, there has been an increase in the number of students willing to get a diploma in their postgraduate fields. Thus, it became necessary to conduct research on the written abstracts as they are considered the essential section of the written theses that will give the reader an idea of the value of the whole dissertation. Hence, to draw attention to its importance and highlight the features that must be included in them, abstract analysis becomes more significant at this point. Especially for those writing their M.A. or Ph.D. abstracts, to guide them on what is needed. Any abstract that is going to be written needs systematic and organized work. The absence of these may lead to comprehension problems and may cause less attention. Poorly written abstracts can have unwanted results and may not receive enough credit or be read. To avoid this, what is expected is that a writer should have all the necessary skills to write good abstracts, which should be seen or understood from the moment one looks at the study. Genres and moves should be included and defined so that every reader understands each step clearly without reading the whole research. Genre is a literary term, and genre analysis is a sort of discourse done to check the reliability of communicative purposes. So, it includes an analysis of the style and text. Abstracts as genres have become a key tool for investigators because they offer them a chance to choose the appropriate study for their investigation (Chen and Su, 2011; Yelland, 2011; Piqué-Noguera, 2012; Paré, 2017; Abdollahpour and Gholami, 2019; Anderson et al., 2021; Yu, 2021).

Moreover, genre referring to abstracts means socially known ways of using language. This is because writing practise is done to give the reader a chance to interpret what the reader could be expected based on what they have read in earlier texts (Hyland, 2007). As Kaya and Yağız (2020) state, publishing research articles in English is the main aspect of academic life. Therefore, writing is a challenging job, and surviving in the academic world is demanding. Thus, it needs to be of good quality with all the features. However, if the writer has no awareness of what is required for writing and how to make it more interesting for the readers, the text written can turn into a disappointment. Belcher (2009) states that the abstract is an important part of work because it gives readers an idea of what it contains and whether it is worth reading. To put it simply, an abstract acts as a communication tool revealing the importance of the article and indicating whether reading the article will enrich scholars. Tanko (2017) claims that abstracts are the key tools to declare the outcomes researchers have found in their studies. Moreover, Salager-Meyer (1994) and Hartley (2003) define the abstract as the core of the article and the first part that encounters the readers of the article. For this reason, Hartley and Betts (2009) highlight the importance of abstracts by pointing out the fact that a well-written abstract increases the possibility of being read if it gives enough information about the article.

This study investigates and analyzes the M.A. and Ph.D. students’ theses abstracts written by EFL Turkish students in the ELT department. As we all know, abstracts are a very significant part of articles, and they are the main part of transferring and reporting the writer’s view. The major concern of this issue is probably the poor writing skills of students’. Since the demand for writing abstracts is increasing, it has become more important to focus on the structure of the information, make discourse and do a genre analysis. Therefore, problems that lie beneath this topic will be examined, analyzed, and solved.

This study aims to analyze how M.A. and Ph.D. students at Cyprus International University (CIU) write their thesis abstracts using a genre-based approach and Hyland’s framework for abstract analysis (2000). Specifically, the study aims to classify the patterns of the moves employed in the abstracts of CIU M.A. and Ph.D. theses, identify the obligatory and optional moves in the research abstracts, and determine the linguistic features, specifically the tense of the verb and the voice of the verb. Furthermore, it aims to classify the pattern of the rhetorical moves employed in the theses abstracts and determine the linguistic features used by the researchers regarding the following: (a) tense of the verb and (b) voice of the verb. The following research questions will be answered through the present study:

(1) What are the genre-specific rhetorical features of the abstract sections of M.A. and Ph.D. theses written between the 2009 and 2019 academic years on foreign language education at Cyprus International University?
(2) What types of moves are there in the abstract sections of M.A. and Ph.D. theses written between the 2009 and 2019 academic years on foreign language education at Cyprus International University?
(3) What are the obligatory, conventional, and optional moves identified in the abstract sections of M.A. and Ph.D. theses written between the 2009 and 2019 academic years on foreign language education at Cyprus International University?
(4) What are the linguistic features of the abstract sections of M.A. and Ph.D. theses written between the 2009 and 2019 academic years on foreign language education at Cyprus International University?

## Theoretical Framework

Abstracts are the most important parts of research reports as they determine the value of the whole manuscript. Therefore, as Male (2018: 24) states, “abstracts categorized as an academically written genre containing the rhetorical structure or moves”. Writing abstracts can be more challenging than writing the whole report for students or academicians since it requires an awareness of steps or organization. Furthermore, it has to be written systematically and in good organization. According to Othman (2011), effective abstract writing can be ascribed to many factors. One of the aspects written is organization. Abstracts are important for the growth and prosperity of academics in all fields.

Considering the fact that the English language has become an international language used worldwide, it may carry some obstacles within itself for non-native speakers when they are writing their reports. Especially, when they want to convey their thoughts. This could be one of the reasons Hyland (2016) has pointed out why non-native speakers go through difficulties as the linguistic norms of the target language are different from their mother tongue. Similarly, Brown (2000) has stated that not only writing is a complicated activity but also one needs to have the full competencies.

According to Ren and Li (2011), genre analysis has to be done to be able to write well or to overcome the challenges of academic writing. For this reason, Al-Zubaidi (2012), recommended that second language learners’ should receive extra help in comprehending the content, building academic language, and incorporating language skills. Furthermore, Zhu (2004); Tardy (2005), and Tas (2008) pointed out that in the process of writing academic manuscripts, appropriate style should be given in a discoursal environment. To overcome the writing difficulties in the native language and to develop effective academic writing skills studying the genre, analysis is the best.

Due to the fact that examiners or readers are very busy doing their work, most of them limit their search, and they want to know from the first glance whether the manuscript is worth reading or not (Alhuqbani, 2013). For this reason, according to Kossasih (2018), four reasons make abstracts play a vital role in articles. The first reason is, it gives information that can be easily read or seen. The second reason is that it can guide readers or provide them with a clue as to whether they will finish reading the whole content or not. Third, it gives an outline for readers. Fourth, it offers a summary of the most important ideas and thoughts. According to Walter (2008), abstract means, “a shortened form of a speech, article, book, etc., giving only the most important facts or ideas.” Bhatia (1993) defined it as “a description or factual summary of the much longer report, and is meant to give the reader an exact and concise knowledge of the full article.”

Moreover, Martín-Martín (2005: 20) claims that abstracts are written: “to provide the summary of the content of the accompanying article”. Consequently, they all suggest that research article writers should use a series of rhetorical strategies or move structures, and accordingly, there are some popular generic structures to mention. One of them is Bhatia (1993), suggesting four-move generic structures of abstracts: (1) introducing the purpose, (2) describing the method, (3) summarizing the result, and (4) presenting the conclusion. Another one is proposed by Hyland (2004), which has a five-move generic structure such as introduction, purpose, method, product, and conclusion moves. In parallel with these, Santos (1996) and Swales and Feak (2004) proposed a five-move generic structure that include (1) background, (2) aim, (3) method, (4) results, and (5) conclusion moves.

According to Kossasih (2018), the abstract can be contemplated as a genre. Eggins maintains that “Genre is a staged, goal-oriented purposeful activity in which speakers or writers engage as members of our culture. Thus, recognizing the genre of a text has an important role in identifying ways in which a particular text is similar to, reminiscent of, other texts circulating in the culture” (Eggins, 2004: 45). Hence, if the genre of the text cannot be identified, it can be seen as problematic. According to Niu (2013), the genre is a literary term, and genre analysis is a type of discourse that is believed to be done to check the consistency of communicative purposes. Therefore, it involves stylistic text analysis. Abstracts as genres have become an indispensable tool for researchers because it provides them with a chance to select the right article for their research (Piqué-Noguera, 2012).

**FIGURE 1:**
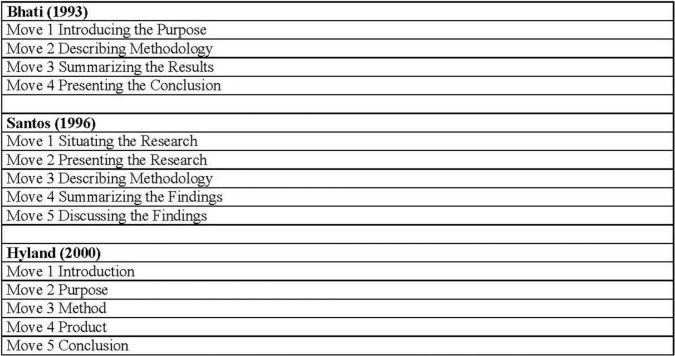
Research article abstracts move models (El-Dakhs, 2018).

In 1990, Swales identified genre analysis as parts that constitute moves and linguistic features such as tense, reporting verbs, and the lexical frequency that help writers write a certain text. Thus, it focuses on ideas and meaning and has a sequence of moves that involves communicative function in each move. Therefore, according to his description and identification, all research articles should first identify the topic, then give a review of the previously written articles as the next move, and detect what is not present in the research reports written earlier as a second move, and state the outline of the goals of the study that has been carried out by writing a summary of the outcomes and stating the results as the last move (Upton and Connor, 2001). In line with Upton and Connor (2001), Ding describes the word move as, “A functional unit in a text, being related to the overall task, which is used to identify the textual regularities in certain genres of writing” (Ding, 2007, 20). Having read many articles on the topic, it has been noticed that many experts have defined the term “move analysis” differently. Yelland (2011: 12) defines move analysis as a “piece of text that is evident in the unified functional meaning of a sentence or group of sentences”. Swales (2004) defines it as a shaper of the overall communicative purpose and the rhetorical structure of the genre. El-Dakhs (2018) explains each move as steps. Moreover, some models have been put forward by Bhatia (1993); Santos (1996), and Hyland (2000). In Bhatia’s version, four moves have been explained, namely, introduction, method, results, and discussion, and this model has been named the IMRD model. In Santos (1996) suggested a new model and put forward five moves, namely, situating the research, presenting the research, describing the methodology, summarizing the findings, and discussing the findings. Finally, in 2000, Hyland gave the final version of the model of moves, which can be detected as similar to Santos’s model because Hyland’s version also included five moves and introduction, purpose, method, product, and conclusion made up the model. Figure 1 demonstrates the three different models that have been explained.

According to Can et al. (2016), moves can be explained or defined as methods that can be used as a guide for the organizing the text to be written. Therefore, all these studies that have been carried out with the aim of evaluating the language of thesis abstracts are to offer different ways and methods of organizing and controlling the language of second language learners’ to prevent discrepancies for communicative purposes. Furthermore, Kanoksilapatham (2007) stated that the move analysis helps the information to be arranged and identifies the type of information that should be included in the text.

This study employs Hyland’s five-move model, examining the moves in thesis abstracts by CIU students. The results of this study can help non-native speakers in writing their abstracts and can contribute to the enrichment of literature in regards to the structure of moves. Moreover, the results are expected to provide deep insight for academicians to improve their writing skills in their future studies.

## Method

This study adopts a content analysis method to examine the rhetorical structure of English thesis abstracts. The corpus is the analysis of a total of 50 bachelor theses abstracts at Cyprus International University. The students were selected randomly. The analysis of thesis abstracts involves examining Hyland’s five-move models, which consist of introduction, purpose, method, product, and conclusion.

As shown in Table 1, Hyland (2000) presents all five moves and their functions. We examined all moves and their functions sequentially in our analysis.

**TABLE 1:** Hyland’s (2000) move model.

Move	Function
Introduction	Establishes the context of the manuscript and motivates the research or discussion
Purpose	Indicates purpose, thesis or hypothesis, outlines the intention behind the manuscript
Method	Provides information on design, procedures, assumption, approach, data, etc.
Product	States main findings or results, the argument, or what was accomplished
Conclusion	Interprets or extends results beyond the scope of the manuscript, draws inferences, points to applications or wider implications

## Findings

To examine abstracts more easily, all 50 abstracts were numbered separately. Hyland’s (2000) model was employed for the analysis. The reason for choosing this model is the fact that it includes five moves, namely, introduction, purpose, methods, product, and conclusion, and his model was used widely in many other research studies. Hyland’s model has been accepted as the most influential in addressing the rhetorical moves in abstracts compared to other suggested models by Santo and Bhatia. In the analysis of move, identification, occurrence, patterns, and the use of tense and voice of moves were also examined. Moreover, the analysis was based on the content or function of the text, and the frequency was used to classify the number of move occurrences in the abstracts. The frequency of occurrence was first recorded and then noted in the tables.

Table 2 lists the frequency of moves found in the abstracts, and as it can be seen from the table, purpose and method have the highest percentages, and conclusion has the lowest percentage. It also shows that only (20%) of these abstracts include complete Hyland’s five moves which are: introduction, purpose, method, product, and conclusion. It has been noticed that most English thesis abstracts (70%) comprised only four rhetorical moves with the absence of a conclusion move. It can be said that all abstracts did not have the conclusion move. The absence of some rhetorical moves in English thesis abstracts may cause communication problems with the readers. Moreover, the readers may have difficulty comprehending the text, and may fail to read it further. This may be a drawback for researchers if their research is not read and recognized in the academic community.

**TABLE 2:** The frequency of moves found in the abstracts.

Moves	Corpus n (50)
Introduction (I)	37 (74%)
Purpose (P)	48 (96%)
Method (M)	46 (92%)
Product (P)	44 (88%)
Conclusion (C)	25 (50%)

In Table 3, the results for tense verb frequency in each move in the abstracts are presented. According to the results obtained, it can be seen that most of the moves in thesis abstracts were written in the present tense. The results showed that the present tense was used more than the past tense in the introduction, purpose, and conclusion in English abstracts. However, the past tense was used more with higher percentages in method and product move. It can also be seen that the future tense was only used in the conclusion move with a low percentage (12%), and it cannot be seen in the other moves.

**TABLE 3:** Verb tense frequency in each move in the abstracts.

Tense of moves	Corpus n (50)
Introduction (I)	
Present	30 (81%)
Past	7 (19%)
Purpose (P)	
Present	43 (90%)
Past	5 (10%)
Method (M)	
Present	6 (13%)
Past	40 (87%)
Product (P)	
Present	15 (34%)
Past	29 (66%)
Conclusion (C)	
Present	16 (64%)
Past	6 (24%)
Future	3 (12%)

In Table 4, the findings showed that the active voice was preferred in the introduction, purpose, product, and conclusion moves. Nevertheless, the passive voice was used in the method move with a higher percentage compared to the active voice. Furthermore, it is possible to say that a mixture of active and passive voices was used in all moves in the analyzed abstracts.

**TABLE 4:** The voice used in the analyzed abstracts.

Moves	Corpus n (50)	
	Active	Passive
Introduction (I)	21 (57%)	16 (43%)
Purpose (P)	43 (90%)	5 (10%)
Method (M)	21 (46%)	25 (54%)
Product (P)	41 (93%)	3 (7%)
Conclusion (C)	19 (76%)	6 (24%)

In the identification process of move analysis, the belief in rhetorical function was vital for the analysis of RA abstracts to investigate move frequency, move pattern, and the use of tense and voice. To ensure the reliability of this research, coding was used. The Kanoksilapatham’s (2015) criterion for the classification of the frequency of occurrence of each move was employed as the cut-off point.

## Discussion

As shown in Table 1, all five moves related to Hyland’s (2000) model have been presented. In Table 2, the frequency of each move differed slightly. The purpose move has been found to have the highest frequency and percentage (96%), followed by the method (92%), product (88%), introduction (74%), and conclusion moves (50%). According to the data obtained, the conclusion move with the least frequency and percentage was an optional move as it was not mentioned in most abstracts. Many researchers preferred not to include this move in their abstracts as this showed they did not give enough importance to it. However, the purpose, method, and product moves were similar in their frequency, but the introduction and conclusion moves seemed to be different, with the least occurring frequency and having the least percentages. The purpose move was the most dominant in all the abstracts that have been examined in this study. The high frequency found in the moves of purpose, method, and product implies that the researchers were aware of the importance of these three moves, whereas the least frequency found in the introduction and conclusion moves demonstrates that some researchers were not aware of the importance of establishing the context of the manuscript and motivating the research or discussion and interpreting or extending the results beyond the scope of the manuscript, drawing inferences, pointing to applications, or suggesting wider implications. The writers had a tendency to begin their abstracts with a purpose move and end the abstracts without drawing references to the field by providing no further suggestions on how to improve their studies in the future. This finding indicates that the writers of this corpus regard the background, method, and significance of the study as more important. Since there is no previously written similar research on this issue, this can be interpreted as the writers’ are lacking rhetorical knowledge on the other two moves (introduction and conclusion), or perhaps they do not attach any importance to mentioning them.

As shown in Table 3, the most frequent verb tense in all the five moves was the present tense. However, the most frequent pattern can be seen in the purpose move with a percentage of 90%. Only in the method move past tense was more frequent with a percentage of 87%. The most frequent patterns were in introduction move (a) Pr-P, purpose move (b) Pr-P, method move (c) P-Pr, product move (d) P-Pr, and conclusion move (e) P-Pr-F. It can be seen that the present voice was the most frequently preferred structure, and only in the conclusion move, the future tense was preferred only in three abstracts among 50 manuscripts to give further implications on the study. According to Table 3, in the introduction, purpose and conclusion moves present tense was used more frequently and to categorize this present simple and present continuous, and present perfect tenses were the most frequently seen. In the product move, the use of past tense was seen to be more than the present tense. Finally, in the conclusion move, from the findings, it can be understood that all tenses are used, including past, present, and future, however, the further findings reflect that the present tense was used more, followed by past and future tense. The differences in tenses usage and their frequency were in the method and product moves. There was no future tense used in the other moves, while 12% of the ELT abstracts were written in the future form. For the method move, only 13% of the abstracts were written in the present form, while 87% were written in the past tense. Thus, we can say that the most frequent tense used in most moves was present simple, while the past tense was the second most frequent and the future tense was found to be the least frequent in three abstracts only to present the conclusion move. However, other previous studies by Zhang et al. (2012) and Suntara and Usaha (2013) stated that the most dominant tense was the past tense in the studies they have carried out.

When our study is compared with other studies by Tseng (2011) and Alhuqbani (2013), it has been found that they both included the same similarities and differences in tenses usage. This means that in the introduction, purpose, and conclusion moves, they tended to use the present simple tense, whereas in our study, introduction, purpose, product, and conclusion moves present tense was mostly used, but the method and product moves were different in the tense usage because past tense was used more which is similar to Alhuqbani’s and Tseng’s findings. Tseng found that in method and product moves, past tense usage was more dominant, which is similar to our findings. However, Zhang et al. (2012) suggested that in their findings, present tense was not seen in the method move in the abstracts he examined. This implies that there are variations in the methodology part of the writings of research manuscripts’ abstracts.

In Table 4, the findings showed that in general, active voice usage was mostly used in all moves. Especially, in the purpose move, the active voice was used by 90% with the highest percentage, while the passive voice was used by 10%. The second most frequent choice was a mixture of active and passive voices that occurred in the method move. This was similar to Zhang et al. (2012) findings, which stated that active voice was more frequent than passive voice. On the contrary, Tu and Wang (2013) revealed that passive voice was the most frequently used in the RA abstracts they have examined. Moreover, Hanidar (2016) also mentioned that writers prefer to use the passive form more when they are presenting the procedure of their research and stating their findings. Nevertheless, in our findings, a combination of the active and passive voices was used, which indicates that most writers tend to develop their abstracts directly rather than using an indirect style. Only in the product move, the passive voice seem to be the least frequent with the lowest percentage (7%).

Although there have been a lot of studies conducted on abstract writing by both native and non-native speakers of English, my research is different from the previous studies due to the fact that only abstracts written by Turkish students were analyzed. The reason for conducting this research only on Turkish students is to identify strengths and weaknesses in the moves of the abstracts, as there is a wealth of literature available on native students. We believe that this study will contribute to the field, improve the current literature on the topic, and provide a significant step by examining the rhetorical structure of Turkish abstracts within the framework of Hyland’s (2000) five-move pattern. The findings of a study conducted by Çandarlı (2012) showed that all abstracts include the introduction move. The reason for this could be the move pattern he followed (IMRC), in which the purpose move had to be stated in the introduction move. In the study carried out by Al-Khasawneh (2017), it was found that in the examined abstracts by native and non-native speakers of English, three moves (introduction, purpose, and product) were available, which implies that both abstract writers are aware of the importance of the moves in their abstracts. However, the only difference was detected in the abstracts of native writers because they included introduction and conclusion moves more than the non-natives. It is believed that this study can help students and novice writers, especially those from non-English backgrounds to facilitate their successful acculturation into their disciplinary community. Another study conducted by Çakır and Fidan (2015) is believed to raise students’ awareness and help them choose suitable moves to fulfill their aims. Moreover, it is believed that their study will have important implications for the future. The findings of a study done by Kaya and Yağız (2020) are assumed to help authors in this field be familiar with abstract writing conventions. Also, the results are believed to benefit the production of academic writing materials for scholars and academic writing courses. Since it is a comparative study, the results would help non-natives be aware of the conventions of academic writing and guide them throughout the process involved in global research. However, the results of Ashofteh et al. (2020) demonstrate that non-native speakers use more hedges and are more tentative in their abstracts which shows that they leave more space for opposing views in their claims. Furthermore, Saidi and Khazaei’s (2021) study is believed to be used in teaching academic writing to graduate students in English for academic purposes and to help them present their findings globally.

Generally, the authors’ aim in conducting research in this field is that they believe it will be beneficial for beginner writers. The suggestions and recommendations and the findings of the results will guide them to produce better academic reports by following the rules to develop writing skills. In this regard, it would be appropriate to say that this research is expected to provide similar pedagogical implications.

## Conclusion

This study has been carried out to investigate the rhetorical structure of English RA abstracts in ELT theses. Five moves have been identified and analyzed. The present tense and active voice were the most chosen and frequently occurring. As a result of this, the past tense, present perfect tense, and passive voice were seldomly used. Moreover, the findings of this study are presented in a descriptive style since all the results are discussed. For this reason, the authors who will be writing manuscripts in the field of English language teaching should consider these findings and develop their abstracts accordingly. The benefit of this study would be to apply what is useful and needed for the implementation of pedagogical practice. Writing abstracts can be helpful for the development of teaching materials and thesis manuscripts, and with the correct guidance, non-native writers or graduate students who are in the process of developing their careers can be helped to solve their writing problems and organize their work in five moves. Moreover, these five moves would help the development of English abstracts for conference presentations or publications. It is believed that once writers gain a full understanding of grammatical and rhetorical features, they will be able to write their abstracts more effectively. Furthermore, the findings regarding the tense and voice usage presented in this study would be a guide to offer the limitations and drawbacks when writing abstracts. Thus, these restrictions should be considered when carrying out move analysis studies in the future. Peacock (2002) claims that move structures should be taught to non-native speakers and novice writers to help them to be able to write the abstract sections of their research correctly. However, this study is limited since it only focuses on one section, which is abstracts. Likely, another limitation of this study could be the small sample size due to its restriction to 50 abstracts, with the result that it can be generalized to all ELT thesis abstracts. Future studies can focus on the large scale of samples, considering all the suggestions and recommendations made in this research.

## Data Availability Statement

The original contributions presented in the study are included in the article/supplementary material, further inquiries can be directed to the corresponding author/s.

## Author Contributions

SS and BM contributed equally to the manuscript generation, writing process, and approved the submitted version.

## Conflict of Interest

The authors declare that the research was conducted in the absence of any commercial or financial relationships that could be construed as a potential conflict of interest.

## Publisher’s Note

All claims expressed in this article are solely those of the authors and do not necessarily represent those of their affiliated organizations, or those of the publisher, the editors and the reviewers. Any product that may be evaluated in this article, or claim that may be made by its manufacturer, is not guaranteed or endorsed by the publisher.
